# Material Extrusion of Structural Polymer–Aluminum Joints—Examining Shear Strength, Wetting, Polymer Melt Rheology and Aging

**DOI:** 10.3390/ma15093120

**Published:** 2022-04-26

**Authors:** Stephan Bechtel, Rouven Schweitzer, Maximilian Frey, Ralf Busch, Hans-Georg Herrmann

**Affiliations:** 1 Chair for Lightweight Systems, Saarland University, Campus E3 1, 66123 Saarbrücken, Germany; rouven.schweitzer@gmx.de (R.S.); hans-georg.herrmann@izfp.fraunhofer.de (H.-G.H.); 2 Chair of Metallic Materials, Saarland University, Campus C6 3, 66123 Saarbrücken, Germany; maximilian.frey@uni-saarland.de (M.F.); r.busch@mx.uni-saarland.de (R.B.); 3 Fraunhofer Institute for Nondestructive Testing IZFP, Campus E3 1, 66123 Saarbrücken, Germany

**Keywords:** structural joints, aging, material extrusion, additive manufacturing, ABS, PETG, PLA, aluminum, polymer rheology, thermal joining

## Abstract

Generating polymer–metal structures by means of additive manufacturing offers huge potential for customized, sustainable and lightweight solutions. However, challenges exist, primarily with regard to reliability and reproducibility of the additively generated joints. In this study, the polymers ABS, PETG and PLA, which are common in material extrusion, were joined to grit-blasted aluminum substrates. Temperature dependence of polymer melt rheology, wetting and tensile single-lap-shear strength were examined in order to obtain appropriate thermal processing conditions. Joints with high adhesive strength in the fresh state were aged for up to 100 days in two different moderate environments. For the given conditions, PETG was most suitable for generating structural joints. Contrary to PETG, ABS–aluminum joints in the fresh state as well as PLA–aluminum joints in the aged state did not meet the demands of a structural joint. For the considered polymers and processing conditions, this study implies that the suitability of a polymer and a thermal processing condition to form a polymer–aluminum joint by material extrusion can be evaluated based on the polymer’s rheological properties. Moreover, wetting experiments improved estimation of the resulting tensile single-lap-shear strength.

## 1. Introduction

Additive manufacturing (AM) offers huge technical potential, especially with regard to sustainable and customized solutions [1,2]. One of the most common AM technologies is material extrusion (ME), where the extruded material (i.e., polymer) is dosed in a targeted way to generate a three-dimensional part layer-by-layer—cf. DIN EN ISO/ASTM 52900-2017. This process is also referred to as fused deposition modeling^®^ (FDM^®^) or fused filament fabrication (FFF). Lightweight systems in particular often require joining multiple components or materials due to size limitations of the production process or locally varying demands concerning functionality and costs [3,4]. Recently, Frascio et al. [3] emphasized the need for suitable joining concepts with regard to additive manufactured adherends and adhesives. One approach, examined in this work, is to use the polymer processed by ME as (hotmelt) adhesive, resulting in a smooth transition between the joint and the subsequently generated polymer part. Challenges exist primarily with regard to reliability and reproducibility of the additively generated joints. For industrial applications, lack of knowledge about joint degradation and aging resistance represents the key issue regarding this technique.

**Figure 1:**
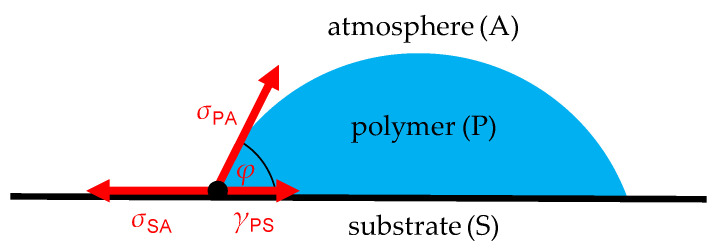
Polymer drop on substrate surface.

From adhesive technology, wetting is known to be a crucial factor to buildup adhesive interactions between the substrate surface and the adhesive. Habenicht [5] distinguished between mechanical and specific adhesion. The former corresponds to a form fit on the microscale, while the latter results from chemical and physical adsorption of the macromolecule functional groups onto the adherent surface. Due to the absence of highly chemically reactive functional groups in the thermoplastics used for ME, only physical adsorption, e.g., Van der Waals forces, dipole–dipole-interactions or H-bonding, is relevant. Both mechanical and specific adhesion require the polymer melt to penetrate into the substrate’s surface texture to establish a form fit or short-range interactions, respectively. Hence, wetting is an indispensable precondition for strong polymer–metal bonding [6]. In thermodynamic equilibrium, the wetting angle, *φ*, results from the interfacial tension between polymer and substrate, *γ*_PS_, polymer and atmosphere, *σ*_PA_, and substrate and atmosphere, *σ*_SA_, according to Young’s equation (cf. Equation (1)). The interfacial tension is defined as the mechanical work that needs to be applied to increase the interfacial area by 1 cm^2^. Figure 1 shows a polymer drop on a metal surface where the interfacial tensions are in equilibrium in a vectorial manner. Adhesion is promoted by proper adherent surface preparation, which forms interlocking structures, increases the real surface and generates or exposes highly energetic surface functional groups.

(1) $$\cos\left(\varphi \right)=\frac{\sigma_{\mathrm{SA}}-\gamma_{\mathrm{PS}}}{\sigma_{\mathrm{PA}}}$$

The work of adhesion, which is defined as the mechanical work required to separate two phases with a contact area of 1 cm^2^, can be calculated according to the Young–Dupré equation (cf. Equation (2)). Although wetting is crucial to buildup the adhesive interactions, it is generally not possible to predict the strength of an adhesive joint solely based on the wetting conditions (Equation (2)) [5].

(2) $$W_{A}=\sigma_{\mathrm{PA}}+\sigma_{\mathrm{SA}}-\gamma_{\mathrm{PS}}=\sigma_{\mathrm{PA}}\cdot \left[1+\cos\left(\varphi \right)\right]$$

In general, clean metallic and oxidic surfaces possess a high surface tension, $\sigma_{\mathrm{SA}}$, and are wetted properly by liquids with a low surface tension, such as polymers—$\sigma_{\mathrm{PA}}$ [7]. However, Equation (2) does not account for differences between the advancing and receding angle or for kinetically ruled processes. In thermal equilibrium, liquids with an elevated viscosity, such as polymer melts, wet the substrate gradually. The dynamics of wetting result in viscous friction in the drop and molecular adsorption and desorption processes at the contact line [8,9]. As a result, the contact angle decreases continuously until equilibrium is reached. Depending on the viscosity of the polymer melt, this process takes some 10 min (cf. e.g., [10]). Hence, wetting dynamics depend on polymer melt viscosity, which usually decreases with increasing shear rate and temperature [11]. Furthermore, the polymer melt surface tension, $\sigma_{\mathrm{PA}}$, decreases with increasing temperature [12]. Hence, increasing the temperature promotes wetting in terms of thermodynamic equilibrium (cf. Equation (1)) and dynamics. Besides the polymer, the type of metal and its surface condition also affect wetting and joint strength due to differences in physical adsorption, interfacial tension (*γ*_PS_ and *σ*_SA_), wetting dynamics and thermally induced internal stresses [5]. While the former two mainly depend on the substrate’s surface functional groups, the latter two depend on the thermal conductivity and expansion coefficient, respectively.

Das et al. [13] emphasized the importance of polymer melt rheology in the context of ME. On the one hand, proper extrusion, wetting and interdiffusion between the adjacent traces is promoted by a low viscosity, *η*, and a high loss factor, tan(*δ*). On the other hand, geometric accuracy and dimensional stability require high viscosity and a low loss factor. The loss factor, tan(*δ*) = *G*″/*G*′, equals the ratio of loss modulus, *G*″, and storage modulus, *G*′, and specifies whether the polymer melt behaves dominant elastic (tan(*δ*) < 1) or dominant viscous (tan(*δ*) > 1). Based on the local thermal history and the viscoelastic polymer melt properties, some authors [14,15,16] derived a polymer healing degree or an effective weld time between adjacent layers in order to predict the interfacial strength. The underlying relaxation phenomena were correlated with, e.g., the reptation time *t*_d_. This time corresponds to a long-range relaxation or diffusion, respectively, of a macromolecule and can be calculated according to Equation (3) based on the zero shear viscosity, $\eta_{0}$, and the plateau modulus, $G_{N}^{0}$ [17]. Another characteristic relaxation time is the rouse relaxation time, *t*_ro_, which corresponds to short-range relaxations at the chain ends and equals the crossover time (cf. Equation (4)) [17].

(3) $$t_{d}=\frac{12\cdot \eta_{0}}{\pi^{2}\cdot G_{N}^{0}}$$

(4) $$t_{\mathrm{ro}}=t{|}_{G\prime =G\prime \prime \;\vee \;\tan\left(\delta \right)=1}$$

For hotmelts, adhesion interface performance is known to be highly sensitive to local temperature management during application. Due to their high thermal conductivity, Habenicht et al. [6] suggested preheating of metal substrates to the melting temperature, *T*_m_, of the hotmelt prior to the joining process. Accordingly, Amancio-Filho and Falck [18] specified substrate temperatures in between the extrusion temperature, *T*_e_, and the crystallization temperature, *T*_c_, of the polymer processed by ME to optimize polymer–metal bonding. However, in their recent publications regarding the Addjoining^®^ process, Falck et al. [19,20] applied a primer onto the aluminum surface before the actual ME joining process was carried out at significantly lower substrate temperatures. Using carbon fiber reinforced polyamide 6 (CF-PA6) instead of acrylonitrile–butadiene–styrene copolymer (ABS) resulted in a higher tensile single-lap-shear strength. Chueh et al. [21] generated poly(ethylene terephthalate) (PET)–steel joints by ME of the polymer on a preheated (180 °C) structured metal substrate with undercuts, which was prepared by selective laser melting (SLM). After filling the surface structures with polymer, the ME process was paused to consolidate the polymer–metal interface by pressure and laser stimulation. By increasing substrate and extrusion temperature, Hertle et al. [22] increased the lap shear strength of ME-joined polypropylene (PP)–aluminum samples. The increased contact temperature resulted in improved filling of the microstructures of the electrochemically treated aluminum surface. Dröder et al. [23] joined ABS to surface structured aluminum substrates. Higher surface structures and substrate temperatures resulted in increased tensile single-lap-shear strength. In our previous work [24], we showed the ability of thermographic process monitoring to characterize the ME joining process in terms of wetting and joint strength, as exemplified by poly(lactic acid) (PLA)–aluminum joints. While Herlte et al. [22], Dröder et al. [23] and Bechtel et al. [24] observed a large influence due to substrate temperature, *T*_s_, Falck et al. [19] reported only a minor effect. As Falck et al. applied a primer beforehand, the polymer was not deposited directly on the metal surface during the ME process. Due to the significantly lower thermal conductivity of the primer, substrate temperature dependence was reduced (cf., e.g., [5]). The presented studies all dealt with polymer–metal joints generated by ME; however, in terms of structural joining and industrial application of the joining concept, the following questions arise:

- *Which of the common thermoplastics for ME is most suitable to generate structural polymer–metal joints?* Direct comparison between the studies is not feasible, in particular due to different processing and substrate surface conditions.
- *Can structural polymer–metal joints be generated by ME on “simple” practical relevant metal surfaces (e.g., prepared by grid blasting)?* In the relevant studies, either a primer was applied beforehand or complex surface preparation methods were used. Moreover, none of the studies considers joint degradation.

This article focuses on the generation of structural polymer–aluminum joints by means of ME. Structural joints are characterized by high joint strength (shear strength greater than 7 MPa) and significant aging resistance [7]. Moreover, we examined how joint strength correlates to wetting and polymer (melt) properties for several thermoplastics in order to obtain appropriate thermal processing conditions. Hence, the originality of this work lies in the characterization and evaluation of ME-generated polymer–metal joints in terms of joint strength and aging resistance by taking into account wetting and polymer melt properties.

## 2. Materials and Methods

The hereafter described approach is a continuation of our previous work [24].

### 2.1. Aluminum Substrates

The aluminum substrates were received from the water-jet cutting company RS-Evolution (Saarwellingen, Germany). The deburred substrates were prepared to measure 25 mm by 115 mm from 2 mm thick sheet metal of EN AW-6082-T6. This medium-strength aluminum alloy has excellent corrosion resistance and is typically used for structural parts in, e.g., the transportation sector [25]. The substrates were grit blasted with corundum (Al_2_O_3_), size F150, which was received from Oberflächentechnik Seelmann (Dessau-Roßlau, Germany). Particle size was about 82 µm and laid within standardized range (45–106 µm)—cf. DIN EN 13887-2003. Sandblasting was performed with a ST 800-J (Auer Strahltechnik, Mannheim, Germany) at a pressure of 6 bar, a working distance of 10 cm and an angle of 90° to the surface.

### 2.2. Material Extrusion (ME)

The polymers were processed by a customized ME machine based on a desktop FFF platform (Ender 3, Creality 3D, Shenzhen, China). The extruder was equipped with a water-cooled heatsink, a “volcano”-type hotend and a brass nozzle with an inner diameter, *w*_Po_, of 0.8 mm (all purchased from E3D Online, Oxfordshire, UK). Three thermoplastic polymers, common for ME, were considered:

- Acrylonitrile–butadiene–styrene copolymer (**ABS**), Extrafill™ (Fillamentum, 768 24 Hulín, Czech Republic), yellow-colored filament
- Poly(ethylene terephthalate) glycol comonomer (**PETG**), PolyLite™ (Polymaker, Shanghai, China), transparent filament
- Poly(lactic acid) (**PLA**), Ingeo™ 3D870 (Nature Works, Minnetonka, MN, USA), black-colored filament

ABS, PETG and PLA were extruded at a temperature, *T*_e_, of 240 °C, 220 °C and 200 °C, respectively. Extruder temperature *T*_e_ was chosen based on polymer melt viscosity. During the deposition of the *d*_Po_ = 0.3 mm high layers, the extruder moved at a velocity, *v*_e_, of 10 mm/s. The polymer-specific substrate temperatures, *T*_s0_, of 100 °C, 80 °C, and 60 °C for ABS, PETG and PLA, respectively, were chosen based on the recommended build-plate temperatures in the datasheets [26,27,28]. In the joining and wetting experiments, the substrate temperature, *T*_s_, was varied. If the substrate temperature at a certain layer *i*, *T*_s,*i*_, differed from the polymer-specific substrate temperature, *T*_s0_, it is specified in the corresponding section. Slicing was done with Ultimaker Cura 4.0 (Geldermalsen, The Netherlands).

### 2.3. Rheometry

Rheometry was performed with an Ar2000ex (TA Instruments, New Castle, DE, USA) rheometer using plate–plate configuration. A 1 mm thick polymer disc with a diameter of 25 mm was generated by ME and placed between the plates of the rheometer. After preheating to 200 °C, the disc was carefully compressed by the rheometer plates to prevent the polymer from squeezing out. Using deformation-controlled oscillation rheology, the deformation amplitude was set to 5%. After equalization for 2 min at the set measurement temperature, oscillation sweeps were carried out in the range from *ω* = 2π·10 Hz down to *ω* = 2π·0.01 Hz. In the first measurement cycle, the set temperature ranged from 200 °C to 130 °C for all polymers. In the second cycle, the maximum temperature was set to 250 °C, 240 °C and 220 °C for ABS, PETG and PLA, respectively, while the minimum temperature was kept unchanged at 130 °C. The third cycle was identical to the first cycle.

Rheometry was used to access the thermo–rheological properties of the polymer melts, which are crucial for wetting, cohesive properties [29,30,31] and adhesion interface performance [6]. In total, 5 specimens were tested for each polymer.

Providing that all relevant relaxation phenomena show the same temperature dependence, relaxation times, *t*_i_, and frequency data, *ω*, were shifted with the horizontal shift factor *a_T_* (cf. Equation (5)). Based on this time–temperature superposition [17], single master curves for modulus, G* = *G*′ + *iG*″, and viscosity, η* = *η*′ + *iη*″, at reference temperature *T*_0_ were constructed according to Equation (6). The vertical shift factors, *b_T_* = *T*_0_/*T*, solely depend on measurement temperature, *T*, and reference temperature, *T*_0_, while the horizontal shift factors, *a_T_*, were fit to the Williams–Landel–Ferry equation with the WLF-constants *C*_1_ and *C*_2_ (cf. Equation (7)).

(5) $$t_{i}\left(T_{0}\right)=\frac{t_{i}\left(T\right)}{a_{T}\left(T,T_{0}\right)},\;\;\;\omega \left(T_{0}\right)=a_{T}\left(T,T_{0}\right)\cdot \omega \left(T\right)$$

(6) $$G^{*}\left(T_{0},a_{T}\omega \right)=b_{T}\left(T,T_{0}\right)\cdot G^{*}\left(T,\omega \right),\;\;\;\;\eta^{*}\left(T_{0},a_{T}\omega \right)=\frac{b_{T}\left(T,T_{0}\right)}{a_{T}\left(T,T_{0}\right)}\cdot \eta^{*}\left(T,\omega \right)$$

(7) $$\log\left[a_{T}\left(T,T_{0}\right)\right]=\frac{-C_{1}\cdot \left(T-T_{0}\right)}{C_{2}+\left(T-T_{0}\right)}$$

### 2.4. Wetting

**Figure 2:**
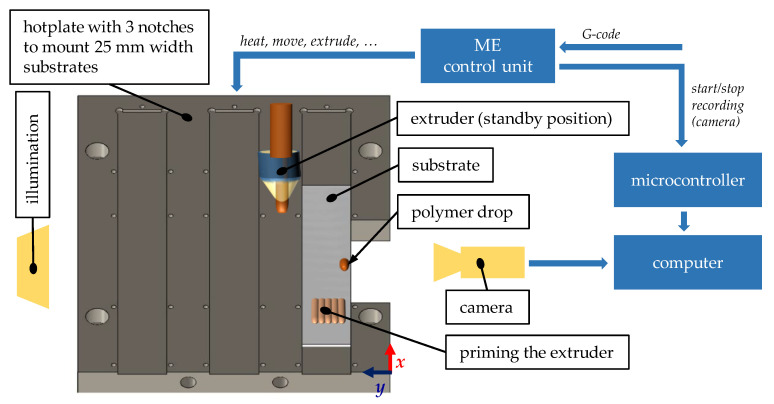
Description of the wetting experiment.

To evaluate wetting behavior, an apparatus for contact angle measurement was added to the ME machine. This apparatus consists of illumination (7W LED spot with diffusor) and a camera (BFS-U3-04S2M (FLIR, Wilsonville, OR, USA), lens: LM50JC (Kowa, Nagoya, Japan)) triggered by the ME machine via specific commands in the G-Code (cf. Figure 2). After preheating the aluminum substrate to the specified first-layer substrate temperature, *T*_s,1_, the primed extrusion nozzle was placed 1 mm above and 2 mm behind the front edge of the substrate and within the field-of-view of the camera. A polymer melt drop with a volume of about 5 µL (referring to the density at room temperature) was extruded before the extrusion nozzle was moved out of the camera’s field-of-view (standby position). Dynamic wetting was recorded for up to 120 min at several substrate temperatures, *T*_s,1_. The highest considered *T*_s,1_ was equal to the polymer specific extrusion temperature *T*_e_. *T*_s,1_ was lowered in increments of 20 °C as long as proper deposition of the polymer melt drop could be achieved.

**Figure 3:**
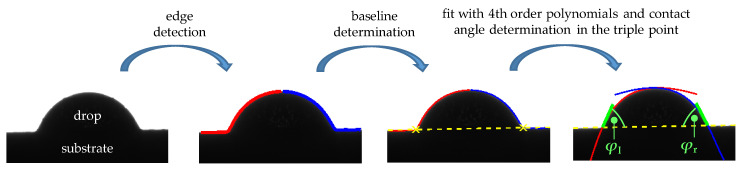
Determination of the contact angle, *φ*, as mean of the left, *φ*_l_, and right, *φ*_r_, contact angle.

Contact angle analysis was done using MATLAB (MathWorks, Natick, MA, USA), as shown in Figure 3 based on the procedure presented by Andersen et al. [32,33]. After image segmentation, edge detection, drop separation, fitting (to the left and right of the apex, 4th-order polynomial) and baseline determination (non-wetted substrate surface on the left and right of the drop), the contact angle was determined at the triple point. This apparent macroscopic contact angle was determined by neglecting surface heterogeneity and roughness. Contact angle measurement was done 3 times for each combination of polymer and substrate temperature.

### 2.5. Mechanical Performance

Tensile tests were carried out using a Kappa 100 DS (ZwickRoell, Ulm, Germany) equipped with a 100 kN loadcell at a controlled ambient temperature of 23 °C. In addition to adhesion interface performance, bulk properties of polymer samples prepared by ME were acquired. All tensile tests were driven-displacement controlled, with 1 mm/min crosshead speed.

Polymer bulk mechanical properties were obtained according to DIN EN ISO 527-2:2012 (type 1A). The extruded tracks were oriented parallel to the loading direction. The polymer was extruded on polyimide-taped aluminum substrates. Due to local excessive tension in the tapered regions [34], these regions were reinforced with an epoxy adhesive (Loctite EA3430, Henkel, Düsseldorf, Germany) to prevent failure outside the measurement area (parallel part). The strain measurement within the parallel part was not affected by the epoxy reinforcement. In total, 6 dog bone tensile test specimens were tested for each polymer.

**Figure 4:**
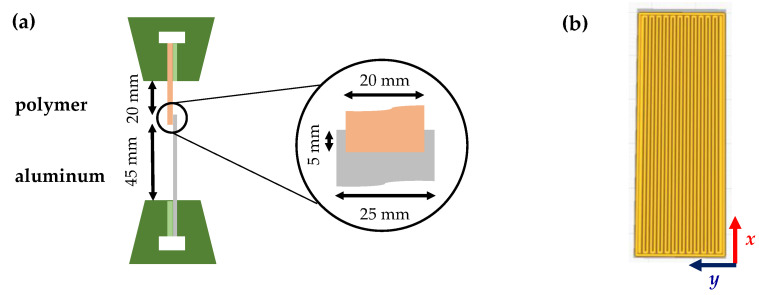
(**a**) Schematic test setup—tensile single lap shear strength; (**b**) ME buildup strategy of the polymer part.

The adhesion interface performance in polymer–aluminum assemblies was evaluated based on ISO 19095 (type B, without specimen retainer). Deviating from the standard, the joint width of the single-lap joints (SLJ) was increased from 10 mm to 20 mm to improve handling. The clamping length was set to 20 mm for the polymer part and 45 mm for the aluminum part to achieve comparable bending stiffness of both adherents. The dimensions and the ME buildup strategy of the polymer part are shown in Figure 4. The tensile single-lap-shear strength, *τ*_SLJ_ = *F*_B_/*A*_J_, was calculated based on the breaking load, *F*_B_, and the joint area, *A*_J_. Failure patterns are classified as polymer part failure (PF, outside the joining area), cohesive failure (CF), adhesive failure (AF) and mixed adhesive and cohesive failure (ACF). The eccentric load path within the joint led to rotation during loading and caused, among other things, additional peel stresses (cf. e.g., [6]). These local stress concentrations caused early joint failure.

The polymer–aluminum joints were prepared by fixing the aluminum substrates on an aluminum hot plate (cf. Figure 2) while extruding the polymer layer-wise on the substrate surface. The part of the polymer adherent beyond the overlap was supported by an aluminum spacer, partially taped with polyimide tape, which was removed after the ME process. The thermal joining process was evaluated by means of the first-layer substrate temperature, *T*_s,1_. For higher layers, the substrate temperature was gradually decreased down to the polymer-specific substrate temperature, *T*_s0_. Table 1 shows the substrate temperature increments for the explored temperature settings. As with wetting, the highest considered *T*_s,1_ was equal to the polymer-specific extrusion temperature *T*_e_. *T*_s,1_ was lowered in increments of 20 °C until the adhesive strength of the joint was too low for handling.

**Figure 5:**
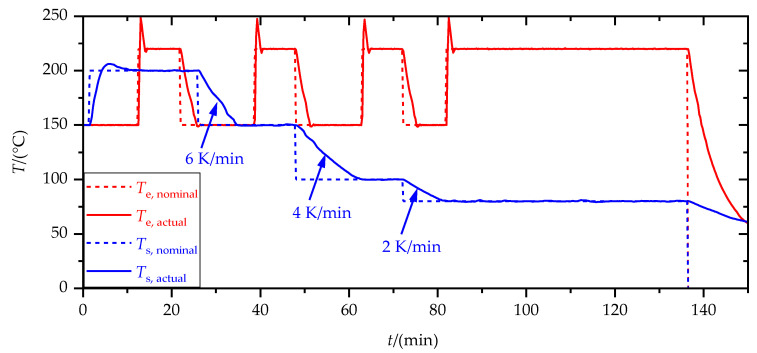
Temperature time profile of the nominal and actual temperature of the extruder, *T*_e_, and the substrate, *T*_s_, during production of an SLJ specimen. Cooling rates represent averaged values.

Figure 5 shows the substrate and extruder temperature profiles during the production of an SLJ specimen in the case of a substrate temperature, *T*_s,1_, of 200 °C and an extruder temperature, *T*_e_, of 220 °C. Sample preparation was carried out on three identical substrates per sequence. In total, 6 specimens were tested for each set of parameters. The fresh SLJ were tested within 5 h after sample production to reduce aging effects.

### 2.6. Aging

Two different moderate environments, which represent real operating conditions, were considered, and are referred to as aging and storing:

- Aging (moist–warm conditions): Darkness, 40 °C and humid air (75% r. h., setup above saturated NaCl solution [35])
- Storing (dry conditions): Darkness, 23 °C and dry air (<8% r. h., setup above silica gel desiccant)

Storing was considered as a reference, in order to separate the effect of internal thermal stresses from hygro–thermo–oxidative aging effects.

SLJs with the highest tensile single-lap-shear strength, *τ*_SLJ_, in the fresh state were aged and stored for up to 100 days. Before mechanical testing, the specimens were acclimatized for at least 1 h at the test climate. Dog bone tensile test specimens were also aged for 100 days to achieve the mechanical polymer bulk properties in the aged state as a reference. For each aging time, 6 samples were tested.

## 3. Results and Discussion

### 3.1. Material Properties

Table 2 gives a summary of selected properties of the aluminum (Al) substrates and polymers used.

Due to the viscoelastic nature of the polymers, their properties were a function of heating, strain rate and temperature. For the technical polymer components investigated, material properties depended, in addition to molecular weight, on additives, such as plasticizers (e.g., [37]). Moreover, the mechanical properties were particularly sensitive to process conditions and buildup strategy (e.g., [38]). While the investigated ABS and PETG were amorphous (no crystallization and melting event), PLA showed crystallization at a cooling rate of 10 K/min. At room temperature, all polymers were in the glassy state, while ABS (*T*_g_ = 99 °C) showed the highest and PLA (*T*_g_ = 58 °C) the lowest glass transition temperature, *T*_g_. The thermal expansion coefficient, *α,* of the polymers was about an order of magnitude higher than that of aluminum. Hence, the thermal joining process resulted in thermally induced interfacial stresses. Internal stress buildup occurred below the glass transition temperature, *T*_g_, in the amorphous phase and below the crystallization temperature, *T*_c_, in the crystalline phase. Consequently, thermally induced interfacial stresses were particularly relevant for PLA and ABS due to crystallization and high glass transition temperature, *T*_g_, respectively. With regard to the sensitivity to the polymer formulation, the process and buildup parameters, and the strain rate, the observed values for elastic modulus, *E*, yield strength, *σ*_y_, and elongation at yield, *ε*_y_, lay in the data range reported in the literature (e.g., [38,39,40,41,42]). The elongation at failure could not be determined reliably, as fracture behavior showed a high variation depending on failure position (parallel part or tapered regions). Moreover, if failure occurred in the tapered regions, strain measurement was inaccurate after the yield point, as failure proceeded outside the measurement marks of the video extensiometry.

#### Polymer Melt Rheology

Viscosity depended on shear rate, $\dot{\gamma }$, and temperature, *T*. In order to determine an appropriate and, in terms of processing viscosity, comparable extrusion temperature for all polymers, the shear rate, $\dot{\gamma }$, of the polymer melt during the extrusion process was obtained by Equation (8) as a function of extruder velocity, *v*_e_, layer height, *d*_Po_, and nozzle diameter, *w*_Po_, as $\dot{\gamma }$ = 48 1/s [43].

(8) $$\dot{\gamma }=\;\frac{32}{\pi }\cdot \frac{v_{e}\cdot d_{\mathrm{Po}}}{w_{\mathrm{Po}}^{2}}$$

**Figure 6:**
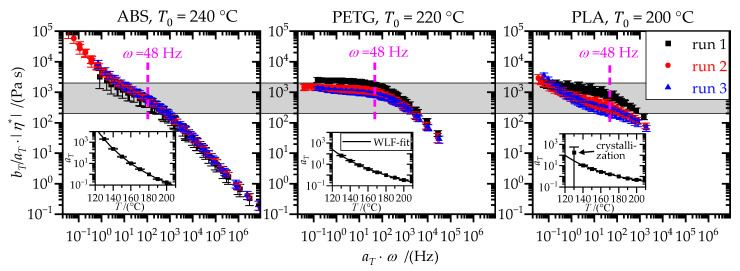
Frequency dependence of the complex viscosity, η*, at a reference temperature *T*_0_ of 240 °C (ABS), 220 °C (PETG) and 200 °C (PLA), respectively, for three subsequent measurement runs. The target viscosity range (gray area) and the shear rate (48 1/s) during the ME process are indicated. Horizontal shift factors, *a_T_*, with corresponding WLF-fit are shown for the first run.

Based on the Cox–Merz rule, $\left|\eta^{*}\left(\omega \right)\right|=\eta \left(\dot{\gamma }=\omega \right)$, which applies to the polymers used [15,44,45], the steady shear viscosity could be approximated based on the oscillatory measurements. For an extrusion temperature *T*_e_ of 240 °C, 220 °C and 200 °C, respectively, ABS, PETG and PLA showed comparable viscosity at *ω* = 48 Hz (cf. Figure 6), which lies in a suitable range for the application of hotmelts and extrusion (i.e., 1 × 10^0^ to 1 × 10^4^ Pa s [5,46]).

**Figure 7:**
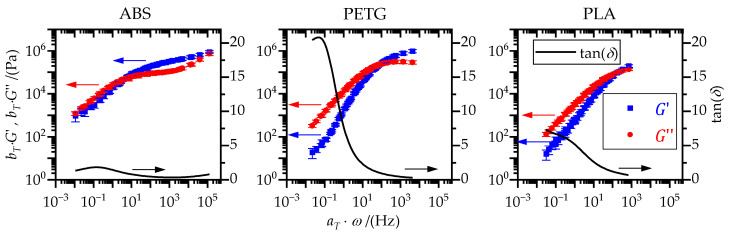
Frequency dependence of storage modulus, *G*′, loss modulus, *G*″, and loss factor, tan(*δ*), at a reference temperature *T*_0_ of 180 °C for the first run. The loss factor was determined based on smoothed master curves of the storage and loss modulus.

Comparing the three polymers in terms of modulus, *G*′ and *G*″, or loss factor, tan(*δ*), revealed pronounced differences, particularly in terms of the loss factor (cf. Figure 7). Especially for low frequencies, the loss factor, tan(*δ*), and thus the viscous character of the polymer melt, was significantly higher for PETG than for PLA and ABS.

For ABS as well as for PLA in the second and third run, no zero shear viscosity, *η*_0_, could be determined, as there was a continuous increase in viscosity for small frequencies, *ω* (cf. Figure 6). The plateau modulus, $G_{N}^{0}$, could not be obtained for PLA because there was not a minimum in *G*′ nor a maximum in *G*″ in the available frequency range (cf. Figure 7). Therefore, the reptation time, *t*_d_, which is calculated based on zero shear viscosity, *η*_0_, and plateau modulus, $G_{N}^{0}$, was not ascertainable for all polymers. Hence, only the rouse relaxation time, *t*_ro_, which could be determined for all polymers, was considered. To account for the differences in viscous character of the polymer melts, the maximum of the loss factor, tan(*δ*)|_max_, in the available frequency range, *ω*, was considered. The maximum of the loss factor, tan(*δ*)|_max_ and the rouse relaxation time, *t*_ro_, are listed in Table 3.

All polymers showed degradation phenomena, resulting in deviations between the subsequent measurement runs. PETG und PLA contain ester groups in their backbones that make them vulnerable to thermally activated, hydrolytic chain scission [47]. Shortening of the macromolecules leads to a reduced relaxation time, *t*_ro_, and viscosity, *η*, (cf. Figure 6 and Table 3). The hydrolysis products are new chain ends with carboxyl-groups [47]. In ABS, the acrylnitril–styrol phase is more stable than the butadiene phase. Thermo–oxidative processes mainly take place at the butadiene double bonds [47]. However, the result is not a chain scission. Instead, subsequent reactions lead to functional groups and cross-linking, which explains the increase in relaxation time, *t*_ro_, and viscosity, *η*, (cf. Figure 6 and Table 3).

Due to heat-induced polymer degradation, the first measurement run is most relevant. However, in the thermal joining and wetting process, the polymers were exposed to similar conditions by means of temperature and atmospheric contact. Hence, the observed degradation phenomena may also be decisive in these processes, in particular with regard to the following aspects:

- ABS and degraded PLA (second and third run) behaved like viscoplastic fluids. These types of fluids do not converge to a zero shear viscosity, *η*_0_, for low shear rates. Instead, the viscosity continuously increases, as the fluids have a yield stress [46]. This is of great relevance for the thermal wetting and joining processes since there is no external force (except gravity) acting on the polymer (melt) after it leaves the extrusion nozzle.
- The carboxyl-(end-) groups resulting from hydrolysis of PETG and PLA can form strong physical bonds (H-bonding) to the metal substrate [5].

### 3.2. Wetting

**Figure 8:**
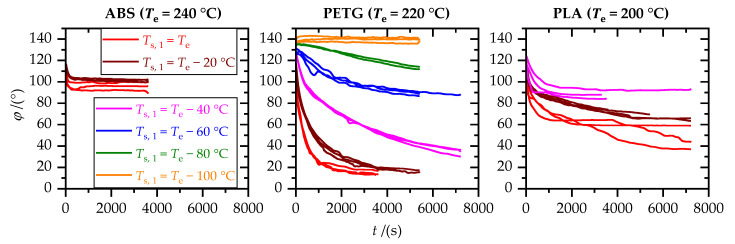
Contact angle, *φ*, between polymer drop and aluminum substrate as a function of wetting time, *t*, for several substrate temperatures, *T*_s,1_.

The temporal change in contact angle, *φ*(*t*), depended on substrate temperature, *T*_s,1_, and polymer (cf. Figure 8). Equilibration took between a few minutes and some hours. According to Table 4, the equilibrium contact angles, *φ*_eq_, lay in the range from 15 to 140°, representing optimal (*φ*_eq_ < 30°) to insufficient (*φ*_eq_ > 90°) wetting conditions [5].

For all three polymers, wetting improved with increasing substrate temperature, *T*_s,1_, which is consistent with temperature dependence of surface tension, *σ*_PA_ [12], and viscosity, *η* (cf. Figure 6). However, there were significant differences between the polymers. PETG showed good wetting, even for substrate temperatures, *T*_s,1_, 40 °C below the extrusion temperature, *T*_e_. Contrary, wetting through ABS was already poor for a substrate temperature, *T*_s,1_, equal to the extrusion temperature, *T*_e_. The polymer-specific wetting behavior is attributed to differences in surface tension, *σ*_PA_, and polymer melt rheology. While the surface tension of the polymer melts and their temperature dependence was not in the scope of this work, the influence of polymer melt rheology on wetting can be discussed. The viscous character of the polymer melts, by means of tan(*δ*)|_max_, was highest for PETG and lowest for ABS (cf. Table 3). Additionally, ABS and degraded PLA behaved like viscoplastic fluids (cf. Figure 6). Since there was no external force acting on the polymer (melt) drop, the yield stress required to start flow may not have been reached. Both the viscoplastic character and the low loss factor, tan(*δ*)|_max_, were adverse for wetting.

### 3.3. Adhesion Interface Performance

#### 3.3.1. Influence of Thermal Processing

**Figure 9:**
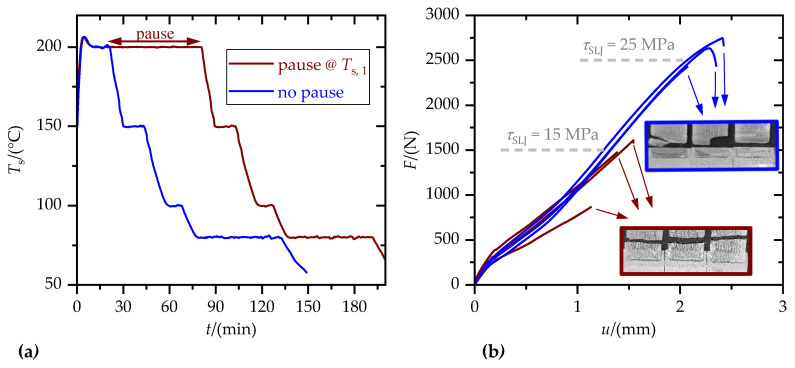
Influence of additional wetting time (1 h pause @ *T*_s,1_) on tensile single-lap-shear strength, *τ*_SLJ_. (**a**) Temperature–time profiles of the actual temperature of the substrate, *T*_s_, during ME of the SLJ; (**b**) Corresponding force–displacement diagrams, *F*(*u*), and SLJ fracture surfaces.

Wetting of the aluminum substrates through the polymer melts depended significantly on wetting time and took up to several hours to reach equilibrium. Therefore, first, the influence of wetting time on tensile single-lap-shear strength, *τ*_SLJ_, was investigated, exemplarily for PETG and *T*_s,1_ = 200 °C (cf. Figure 9). Increasing the wetting time by 1 h decreased *τ*_SLJ_ by about 10 MPa. Hence, contrary to wetting, tensile single-lap-shear strength did not increase with wetting time. Considering the failure patterns reveals that ongoing degradation of the polymer weakened the mechanical bulk properties, leading to polymer part failure (PF). Hence, polymer–metal joints with no additional wetting time were considered.

**Figure 10:**
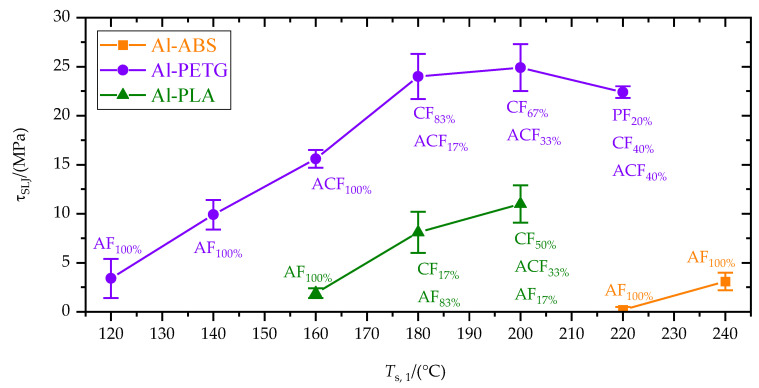
Tensile single-lap-shear strength, *τ*_SLJ_, as a function of substrate temperature, *T*_s,1_. Percentages of the failure patterns: polymer part failure (PF, outside the joining area), cohesive failure (CF), adhesive failure (AF) and mixed adhesive and cohesive failure (ACF) are given.

Figure 10 shows the tensile single-lap-shear strength, *τ*_SLJ_, as a function of substrate temperature, *T*_s,1_. In conjunction with the improved wetting (cf. Table 4), *τ*_SLJ_ increased with increasing substrate temperature. Differences between the polymers were also consistent with wetting. ABS, which wetted the substrate insufficiently (*φ*_eq_ > 90°) at all substrate temperatures, showed the lowest tensile single-lap-shear strength. Contrary, the well-wetting PETG had the highest *τ*_SLJ_. With increasing substrate temperature and tensile single-lap-shear strength, the failure pattern changed from adhesive (AF) over than mixed (ACF) and cohesive (CF) to polymer part failure (PF). For PETG, the shear strength reached a plateau for *T*_s,1_ above 180 °C. This is in accordance with the equilibrium contact angle, *φ*_eq_, which decreased only slightly between 180 and 220 °C (cf. Table 4). Moreover, for *T*_s,1_ greater than 180 °C, cohesive failure (CF) dominated, which means the polymer–metal interface was no longer the weak point. For *T*_s,1_ = 220 °C, there was even a small decrease in *τ*_SLJ_, accompanied by an increase in polymer part failure (PF). Similar to the effect of the additional wetting time (cf. Figure 9), this decrease in *τ*_SLJ_ for the highest substrate temperatures is attributed to degradation of the mechanical polymer bulk properties.

For PETG, tensile single-lap-shear strength, *τ*_SLJ_, decreased for the highest substrate temperatures and long wetting times. Hence, in the thermal ME joining process, two opposing effects in terms of tensile single-lap-shear strength took place. On the one side, wetting improved with increasing substrate temperature and wetting time, but, on the other hand, degradation at high temperatures and long times weakened polymer bulk properties. Wetting time in the ME joining process was just a few minutes (cf. Figure 5) and, hence, significantly shorter than the time required to reach the equilibrium contact angle (cf. Figure 8). However, there was sufficient adhesion formation to reach high tensile single-lap-shear strength and cohesive failure (CF) in the case of PETG and high substrate temperatures (*T*_s,1_ > 180 °C). Consequently, arrangement and orientation of the macromolecules in the wetted area, which is required to form adhesive interactions (i.e., mechanical adhesion and physical adsorption), proceeded much faster than the macroscopic wetting.

#### 3.3.2. Aging Resistance

**Figure 11:**
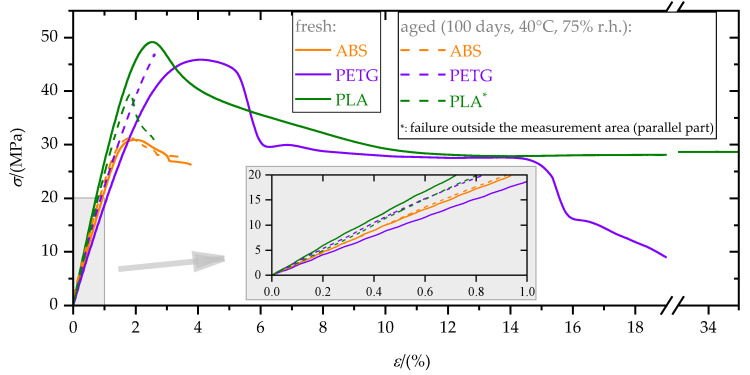
Exemplary stress–strain diagrams, *σ*(*ε*), of the polymers in the fresh and aged state. Graphs in the enlarged section are shifted to (0/0).

First, the effect of aging on the mechanical polymer bulk properties is presented. Figure 11 shows stress–strain diagrams of the polymers in the fresh and aged (100 days) state.

Elastic modulus, *E*, yield strength, *σ*_y_, and elongation at yield, *ε*_y_, are given in Table 5. Aging effects were much more pronounced for PETG and PLA than for ABS. In particular, the yield strength of PLA as well as the elongation at yield of PLA and PETG decreased significantly due to aging. Moreover, while PLA and PETG showed predominantly ductile failure (elongation at failure, *ε*_f_ > 10%) in the fresh state, they broke brittle (*ε*_f_ < 10%) in the aged state. Hence, PLA and PETG became brittle due to aging, which made them more sensitive to notches and local stress concentrations. Reasons for the embrittlement could include the extraction of plasticizers, swelling and hydrolysis (ester groups) [47]. The observed elongation at failure for PLA in the fresh state was higher than usually reported in the literature (e.g., [38]). One explanation for this particularly high elongation at failure is strain crystallization [48]. Due to the low strain rate and material heating caused by plastic deformation, the mechanical glass transition, which depends on strain rate, could be reached, and hence, strain crystallization occurred.

**Figure 12:**
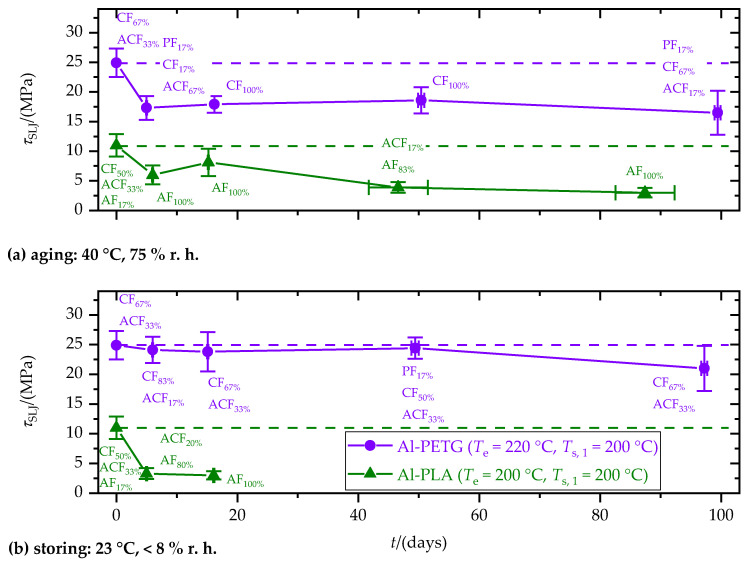
Tensile single-lap-shear strength, *τ*_SLJ_, as a function of (**a**) aging and (**b**) storage time, t. Percentages of the failure patterns polymer: part failure (PF, outside the joining area), cohesive failure (CF), adhesive failure (AF) and mixed adhesive and cohesive failure (ACF) are given.

Due to low *τ*_SLJ_ in the fresh state, Al–ABS-SLJ was not considered for aging and storing experiments. PETG- and PLA-SLJs, with the highest *τ*_SLJ_ in the fresh state, were aged and stored for up to 100 days (cf. Figure 12). The substrate temperature, *T*_s,1_, was set to 200 °C for both polymers.

In the course of aging, *τ*_SLJ_ of Al–PETG-SLJ decreased within 5 days from 25 MPa to 17 MPa and then remained constant. Even in the aged state, no adhesive failure (AF) occurred. Hence, the decrease in *τ*_SLJ_ cannot be attributed to interfacial aging effects. Instead, embrittlement of the polymer component due to aging (cf. Figure 11) in combination with the stress concentrations at the joint edges led to the decrease in *τ*_SLJ_. Storing had no significant effect on tensile single-lap-shear strength, *τ*_SLJ_, of Al–PETG-SLJ.

Tensile single-lap-shear strength, *τ*_SLJ_, of Al–PLA-SLJ decreased continuously from 11 to 3 MPa within 100 days of aging. Additionally, the failure pattern changed in favor of adhesive failure (AF), indicating a weakening of the polymer–metal interface. Contrary to PETG, storing caused an even faster decrease in *τ*_SLJ_. Consequently, the decrease in *τ*_SLJ_ is attributed to thermally induced internal stresses. These stresses built up below the crystallization temperature, *T*_c_, of PLA. Compared to storing, the mobility of the macromolecules increased during aging due to the higher temperature and the plasticizing effect of water. Moreover, swelling counteracted the thermally induced internal stresses. This explains the faster decrease in *τ*_SLJ_ during storing compared to aging.

### 3.4. Correlating Polymer Properties, Wetting and Adhesion Interface Performance

**Figure 13:**
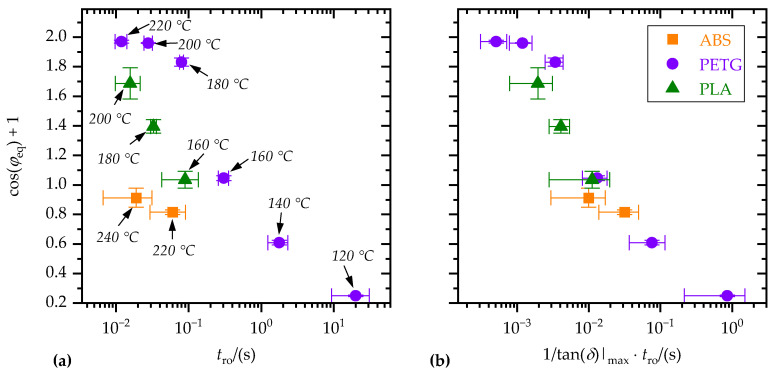
Equilibrium contact angle, *φ*_eq_, as a function of (**a**) Rouse relaxation time, *t*_ro_, and (**b**) Rouse relaxation time, *t*_ro_, and maximum loss factor, tan(*δ*)|_max_. Corresponding substrate temperatures, *T*_s,1_, are indicated.

First, wetting was correlated with the polymer melt properties. Instead of the contact equilibrium angle, *φ*_eq_, the term cos(*φ*_eq_) + 1 was considered, which is proportional to the work of adhesion (cf. Equation (2)). Figure 13a shows cos(*φ*_eq_) + 1 as a function of the rouse relaxation time, *t*_ro_. Temperature dependence of the Rouse relaxation time is given by the horizontal shift factors, *a_T_*. Taking the maximum loss factor, tan(*δ*)|_max_, into account revealed a correlation between polymer melt rheology and wetting, which is almost independent of the polymer material (cf. Figure 13b). According to Figure 13b, wetting improved with decreasing Rouse relaxation time, *t*_ro_, and increasing maximum loss factor, tan(*δ*)|_max_. This is plausible, as *t*_ro_ describes the time dependence of the polymer dynamic processes and tan(*δ*)|_max_ the viscous character of the polymer melt.

**Figure 14:**
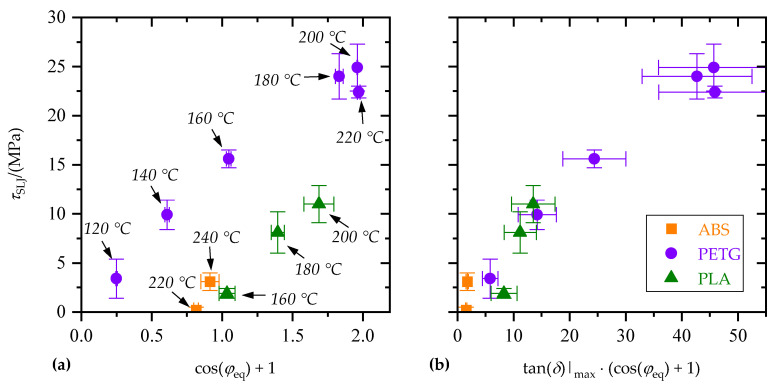
Tensile single-lap-shear strength, *τ*_SLJ_, as a function of (**a**) equilibrium contact angle, *φ*_eq_, and (**b**) equilibrium contact angle, *φ*_eq_, and maximum loss factor, tan(*δ*)|_max_. Corresponding substrate temperatures, *T*_s,1_, are indicated.

Figure 14a shows the tensile single-lap-shear strength, *τ*_SLJ_, as a function of cos(*φ*_eq_) + 1. There were significant differences between the polymers. For a given equilibrium contact angle, *φ*_eq_, single lap shear strength, *τ*_SLJ_, was higher for PETG than for ABS and PLA. Again, by taking the maximum loss factor, tan(*δ*)|_max_, into account, the differences between polymers were significantly reduced (cf. Figure 14b).

**Figure 15:**
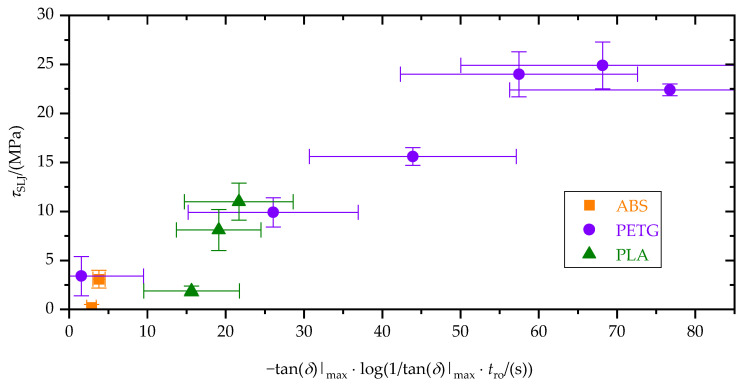
Tensile single-lap-shear strength, *τ*_SLJ_, as a function of the rheologically derived quantities of Rouse relaxation time, *t*_ro_, and maximum loss factor, tan(*δ*)|_max_.

By combining the correlations from Figure 13b and Figure 14b, single-lap-shear strength, *τ*_SLJ_, is shown as a function of the rheologically derived quantities of Rouse relaxation time, *t*_ro_, and maximum loss factor, tan(*δ*)|_max_, in Figure 15.

## 4. Conclusions

This study focused on the generation of structural polymer–aluminum joints by means of ME. Based on the relevant literature [3,19,20,21,22,23,24], the following questions arose:

- *Which of the common thermoplastics for ME is most suitable to generate structural polymer–metal joints?*
- *Can structural polymer–metal joints be generated by ME on “simple” practical relevant metal surfaces (e.g., prepared by grid blasting)?*

These questions were addressed by investigating ME-generated joints between grid-blasted aluminum substrates and the thermoplastics ABS, PETG and PLA as a function of thermal processing (substrate temperature) and aging. For all polymers, tensile single-lap-shear strength increased with increasing substrate temperature. However, there were significant differences between the polymers. For the given conditions and material combinations, PETG was the most suitable to generate structural polymer–metal joints. Appropriate thermal processing conditions for the joining were an extrusion temperature of 220 °C and a substrate temperature of 200 °C. For this case, cohesive failure dominated, and the demands of a structural joint in terms of joint strength and aging resistance were met. Increasing the substrate temperature beyond 200 °C or increasing the time PETG was exposed to the elevated temperatures led to pronounced polymer degradation and reduced joint strength. While storing (dry conditions) had no significant effect on PETG–aluminum joint strength, aging (moist-warm conditions) reduced the tensile single-lap-shear strength due to degradation of the mechanical polymer bulk properties. Hence, this decrease did not result in reduced adhesive strength. Contrary to PETG, ABS–aluminum joints in the fresh state as well as PLA–aluminum joints in the aged state did not meet the demands of a structural joint. PLA–aluminum joint strength decreased faster during storage than aging, which was attributed to internal stresses resulting from the thermal joining process. In particular, crystallization of PLA favored internal stress buildup.

Wetting is known to be crucial to buildup adhesive interactions between substrate surface and adhesive [5]. Polymer melt rheology is a key property in terms of wetting (substrate and adjacent polymer traces) and interdiffusion (between adjacent traces) [6,8,9,13]. Considering this, tensile single-lap-shear strength, *τ*_SLJ_, equilibrium contact angle, *φ*_eq_, and the rheologically derived quantities of Rouse relaxation time, *t*_ro_, and maximum loss factor, tan(*δ*)|_max_, were correlated with each other. For the considered polymers and processing conditions, this study implied that the suitability of a polymer and a thermal processing condition to form a polymer–aluminum-joint by ME could be evaluated based on the polymer’s rheological properties (cf. Figure 15). Moreover, taking into account wetting experiments allowed improved estimation of the resulting tensile single-lap-shear strength, *τ*_SLJ_, (cf. Figure 14b). Remaining deviations between the polymers are attributed to differences in chemical structure and internal stresses. The former is decisive for the types of physical adsorption, and the latter mainly depends on crystallization tendency and glass transition temperature.

To reveal the effect of internal stresses, it would be interesting to vary the crystallization tendency of the polymer or adjust its thermal expansion coefficient by fillers or additives. Moreover, taking into account the temperature-dependent interfacial energies of the polymer melts could improve estimation of the adhesion interface performance based on polymer melt rheology and wetting. This study focused on joining the thermoplastics ABS, PETG and PLA to grit-blasted aluminum substrates. In order to reveal an optimal combination of metal substrate and polymer for the ME joining process, further polymers and metals should be tested. Finally, for a deeper understanding of the ongoing damage mechanism, combining numerical modeling (e.g., [49]) with a characterization of the deformation and failure process by nondestructive testing methods (e.g., [50]) is advisable.

## Figures and Tables

**Table 1:** Substrate temperature at each layer *i*, *T*_s,*i*_, during ME of the polymer part of the SLJ for the explored temperature settings.

Temperature Setting	*T*_s,1_/(°C)	*T*_s,2_/(°C)	*T*_s,3_/(°C)	*T*_s,*i*_*/*(°C), *i* > 4
1	240	150	100	*T*_s0/_(°C)
2	220	150	100	*T*_s0/_(°C)
3	200	150	100	*T*_s0/_(°C)
4	180	150	100	*T*_s0/_(°C)
5	160	150	100	*T*_s0/_(°C)
6	140	100	*T*_s0_/(°C)	
7	120	100	*T*_s0_/(°C)	

**Table 2:** Overview of selected thermal and mechanical properties of the materials used. The melting, crystallization and glass transition temperatures (onset) of the polymers were measured with a DSC3 (Mettler-Toledo, Columbus, OH, USA) at cooling and heating rates of 10 K/min. The thermal expansion coefficients *α* were not obtained for the polymers used; instead, typical ranges for ABS, PETG and PLA, respectively, are given. (*: for Al, the liquidus and solidus temperatures are given).

Property		Al	ABS	PETG	PLA
thermal expansion coefficient	*α*/(10^−6^/K)	23 [25]	108–234 [36]	120–123 [36]	126–145 [36]
density	*ρ*/(g/cm^3^)	2.7 [25]	1.04 [26]	1.25 [27]	1.22 [28]
melting temperature	*T*_m_/(°C)	650 * [25]	amorphous	amorphous	169 ± 0
crystallization temperature	*T*_c_/(°C)	575 * [25]	amorphous	amorphous	119 ± 0
glass transition temperature	*T*_g_/(°C)	-	99 ± 1	75.5 ± 0	58 ± 2
elastic modulus	*E*/(GPa)	70 [25]	2.3 ± 0.1	2.1± 0.1	2.9± 0.1
yield strength	*σ*_y_/(MPa)	280 [25]	30 ± 1	47 ± 1	48 ± 1
elongation at yield	*ε*_y_/(%)		1.8 ± 0.2	4.1 ± 0.1	2.5 ± 0.2
surface roughness	Ra/(µm)				
- blank		0.18 ± 0.02 [24]			
- sandblasted (FEPA 150)		1.9 ± 0.5 [24]			

**Table 3:** Maximum of the loss factor, tan(*δ*)|_max_, and rouse relaxation time, *t*_ro_, at a reference temperature *T*_0_ of 180 °C for three subsequent measurement runs.

Property	ABS, Run 1	ABS, Run 2	ABS, Run 3	PETG, Run 1	PETG, Run 2	PETG, Run 3	PLA, Run 1	PLA, Run 2	PLA, Run 3
tan(*δ*)|_max_	1.9	1.3	1.3	23	25	14	8.0	3.3	2.8
	±0.1	±0.0	±0.0	±5	±6	±3	±1.8	±0.3	±0.2
*t*_ro_ (*T*_0_)/(ms)	1500	2020	2440	80	48	47	33	15	13
	±100	±180	±190	±5	±5	±6	±3	±5	±4

**Table 4:** Equilibrium contact angles, *φ*_eq_, for the explored temperature settings in terms of substrate temperature, *T*_s,1_. (*: Equilibrium was not reached in the wetting experiment. Therefore, *φ*_eq_ was estimated as the last observed contact angle (cf. Figure 8)).

*T*_s,1_/(°C)	*φ*_eq_/(°), ABS	*φ*_eq_/(°), PETG	*φ*_eq_/(°), PLA
240	95 ± 4	-	-
220	101 ± 1	14 ± 2	-
200	-	16 ± 1	46 ± 8
180	-	34 ± 3 *	67 ± 3
160	-	87 ± 1	88 ± 3
140	-	113 ± 1 *	
120	-	139 ± 1	

**Table 5:** Elastic modulus, *E*, yield strength, *σ*_y_, and elongation at yield, *ε*_y_, of the polymers in the fresh and aged (100 days) state.

Property	ABS, Fresh	ABS, Aged	PETG, Fresh	PETG, Aged	PLA, Fresh	PLA, Aged
*E*/(MPa)	2260 ± 50	2320 ± 40	2100 ± 80	2210 ± 80	2880 ± 70	2640 ± 120
*ε*_y_/(%)	1.8 ± 0.2	1.7 ± 0.1	4.1 ± 0.1	2.5 ± 0.3	2.5 ± 0.2	1.8 ± 0.0
*σ*_y_/(MPa)	29.6 ± 1.3	30.3 ± 1.0	46.9 ± 0.9	46.2 ± 3.4	48.1 ± 1.3	41.4 ± 2.0

## Data Availability

The data presented in this study are available on request from the corresponding author.

## References

[1] Bromberger J., Kelly R. (2017). Additive manufacturing: A long-term game changer for manufacturers. The Great Re-Make: Manufacturing for Modern Times.

[2] Ligon S.C., Liska R., Stampfl J., Gurr M., Mülhaupt R. (2017). Polymers for 3D Printing and Customized Additive Manufacturing. Chem. Rev..

[3] Frascio M., Marques E.A.D.S., Carbas R.J.C., da Silva L.F.M., Monti M., Avalle M. (2020). Review of Tailoring Methods for Joints with Additively Manufactured Adherends and Adhesives. Materials.

[4] Klein B., Klein B. (2013). Fügetechniken. Leichtbau-Konstruktion: Berechnungsgrundlagen und Gestaltung.

[5] Habenicht G. (2006). Kleben: Grundlagen, Technologien, Anwendungen.

[6] Habenicht G., Ahner C. (2009). Applied Adhesive Bonding: A practical Guide for Flawless Results.

[7] Ebnesajjad S. (2008). Adhesives Technology Handbook.

[8] Ramiasa M., Ralston J., Fetzer R., Sedev R. (2014). The influence of topography on dynamic wetting. Adv. Colloid Interface Sci..

[9] Lu G., Wang X.-D., Duan Y.-Y. (2016). A Critical Review of Dynamic Wetting by Complex Fluids: From Newtonian Fluids to Non-Newtonian Fluids and Nanofluids. Adv. Colloid Interface Sci..

[10] Welygan D.G., Burns C.M. (1980). Dynamic Contact Angles of Viscous Liquids. J. Adhes..

[11] Elias H.-G. (2001). Makromoleküle: Band 2: Physikalische Strukturen und Eigenschaften.

[12] Adamson A.W., Gast A.P. (1997). Physical Chemistry of Surfaces.

[13] Das A., Gilmer E.L., Biria S., Bortner M.J. (2021). Importance of Polymer Rheology on Material Extrusion Additive Manufacturing: Correlating Process Physics to Print Properties. ACS Appl. Polym. Mater..

[14] Costa S.F., Duarte F.M., Covas J.A. (2017). Estimation of filament temperature and adhesion development in fused deposition techniques. J. Mater. Processing Technol..

[15] Seppala J.E., Hoon Han S., Hillgartner K.E., Davis C.S., Migler K.B. (2017). Weld formation during material extrusion additive manufacturing. Soft Matter..

[16] Bartolai J., Simpson T.W., Xie R. (2018). Predicting strength of additively manufactured thermoplastic polymer parts produced using material extrusion. Rapid Prototyp. J..

[17] Dealy J.M., Read D.J., Larson R.G. (2018). Structure and Rheology of Molten Polymers: From Structure to Flow Behavior and Back Again.

[18] Amancio-Filho S.T., Falck R. (2016). Verfahren zum Herstellen eines schichtförmigen Bauteils. Patent.

[19] Falck R., Dos Santos J.F., Amancio-Filho S.T. (2019). Microstructure and Mechanical Performance of Additively Manufactured Aluminum 2024-T3/Acrylonitrile Butadiene Styrene Hybrid Joints Using an AddJoining Technique. Materials.

[20] Falck R., Goushegir S.M., dos Santos J.F., Amancio-Filho S.T. (2018). AddJoining: A novel additive manufacturing approach for layered metal-polymer hybrid structures. Mater. Lett..

[21] Chueh Y.-H., Wei C., Zhang X., Li L. (2020). Integrated laser-based powder bed fusion and fused filament fabrication for three-dimensional printing of hybrid metal/polymer objects. Addit. Manuf..

[22] Hertle S., Kleffel T., Wörz A., Drummer D. (2020). Production of polymer-metal hybrids using extrusion-based additive manufacturing and electrochemically treated aluminum. Addit. Manuf..

[23] Dröder K., Reichler A.-K., Mahlfeld G., Droß M., Gerbers R. (2019). Scalable Process Chain for Flexible Production of Metal-Plastic Lightweight Structures. Procedia CIRP.

[24] Bechtel S., Meisberger M., Klein S., Heib T., Quirin S., Herrmann H.-G. (2020). Estimation of the Adhesion Interface Performance in Aluminum-PLA Joints by Thermographic Monitoring of the Material Extrusion Process. Materials.

[25] Ostermann F. (2014). Anwendungstechnologie Aluminium.

[26] (2019). ABS Extrafill—Technical Data Sheet.

[27] (2017). PolyLite (TM) PETG—Technical Data Sheet.

[28] (2017). Ingeo Biopolymer 3D870 Technical Data Sheet.

[29] Coogan T.J., Kazmer D.O. (2019). In-line rheological monitoring of fused deposition modeling. J. Rheol..

[30] Coogan T.J., Kazmer D.O. (2019). Modeling of interlayer contact and contact pressure during fused filament fabrication. J. Rheol..

[31] Mackay M.E. (2018). The importance of rheological behavior in the additive manufacturing technique material extrusion. J. Rheol..

[32] Andersen N.K., Taboryski R. (2017). Drop shape analysis for determination of dynamic contact angles by double sided elliptical fitting method. Meas. Sci. Technol..

[33] MATLAB Central File Exchange. Drop Shape Analysis—Fit Contact Angle by Double Ellipses or Polynomials. https://www.mathworks.com/matlabcentral/fileexchange/57919-drop-shape-analysis-fit-contact-angle-by-double-ellipses-or-polynomials.

[34] Gordelier T.J., Thies P.R., Turner L., Johanning L. (2019). Optimising the FDM additive manufacturing process to achieve maximum tensile strength: A state-of-the-art review. RPJ.

[35] Wexler A., Hasegawa S. (1954). Relative humidity-temperature relationships of some saturated salt solutions in the temperature range 0° to 50 °C. J. Res. Natl. Bur. Stand..

[36] (2021). Ansys GRANTA EduPack.

[37] Farah S., Anderson D.G., Langer R. (2016). Physical and mechanical properties of PLA, and their functions in widespread applications —A comprehensive review. Adv. Drug Deliv. Rev..

[38] Goh G.D., Yap Y.L., Tan H.K.J., Sing S.L., Goh G.L., Yeong W.Y. (2020). Process–Structure–Properties in Polymer Additive Manufacturing via Material Extrusion: A Review. Crit. Rev. Solid State Mater. Sci..

[39] Vidakis N., Petousis M., Velidakis E., Liebscher M., Mechtcherine V., Tzounis L. (2020). On the Strain Rate Sensitivity of Fused Filament Fabrication (FFF) Processed PLA, ABS, PETG, PA6, and PP Thermoplastic Polymers. Polymers.

[40] Algarni M., Ghazali S. (2021). Comparative Study of the Sensitivity of PLA, ABS, PEEK, and PETG’s Mechanical Properties to FDM Printing Process Parameters. Crystals.

[41] Chacón J.M., Caminero M.A., García-Plaza E., Núñez P.J. (2017). Additive manufacturing of PLA structures using fused deposition modelling: Effect of process parameters on mechanical properties and their optimal selection. Mater. Des..

[42] Vairis A., Petousis M., Vidakis N., Savvakis K. (2016). On the Strain Rate Sensitivity of Abs and Abs Plus Fused Deposition Modeling Parts. J. Mater. Eng. Perform..

[43] Mackay M.E., Swain Z.R., Banbury C.R., Phan D.D., Edwards D.A. (2017). The performance of the hot end in a plasticating 3D printer. J. Rheol..

[44] Quintans J. (1998). Rheological Characterization of Unmodified and Chemically Modified Poly (Ethylene Terephthalate) Resins. Master’s Thesis.

[45] Bagheriasl D., Carreau P.J., Riedl B., Dubois C., Hamad W.Y. (2016). Shear rheology of polylactide (PLA)–cellulose nanocrystal (CNC) nanocomposites. Cellulose.

[46] Hepperle J., Kohlgrüber K., Bierdel M. (2008). Rheological properties of polymer melts. Co-Rotating Twin-Screw Extruder: Fundamentals, Technology, and Applications.

[47] Ehrenstein G.W., Pongratz S. (2007). Beständigkeit von Kunststoffen.

[48] Zhang X., Schneider K., Liu G., Chen J., Brüning K., Wang D., Stamm M. (2011). Structure variation of tensile-deformed amorphous poly(l-lactic acid): Effects of deformation rate and strain. Polymer.

[49] Campilho R.D.S.G., Banea M.D., Neto J.A.B.P., Da Silva L.F.M. (2012). Modelling of Single-Lap Joints Using Cohesive Zone Models: Effect of the Cohesive Parameters on the Output of the Simulations. J. Adhes..

[50] Summa J., Becker M., Grossmann F., Pohl M., Stommel M., Herrmann H.G. (2018). Fracture analysis of a metal to CFRP hybrid with thermoplastic interlayers for interfacial stress relaxation using in situ thermography. Compos. Struct..

